# Reimagining health services provision for neglected groups: The “personalization from below” phenomenon

**DOI:** 10.3389/fsoc.2023.1052215

**Published:** 2023-02-02

**Authors:** Anna Berti Suman, Nils B. Heyen, Marina Micheli

**Affiliations:** ^1^The European Commission Joint Research Centre, Ispra, Italy; ^2^Fraunhofer Institute for Systems and Innovation Research ISI, Karlsruhe, Germany

**Keywords:** health, public services, personalization, citizen science, data altruism, data governance

## Abstract

How can data-driven citizen science activities supporting health research and services provision meet the needs of unrepresented and neglected groups through increased personalization? In this short Perspective, we explore “personalization from below” as a concept designating forms of citizen science-based data altruism that specifically push for and enact a different understanding of both health services and personalization. We develop the argument that such phenomenon taking place outside “institutionalized” health-related practices could make health services provision more inclusive of values that matter to people. We contextualize instances of “personalization from below,” discuss related data governance models and alternative public health interventions, and conclude by outlining three key arguments in favor of “personalization from below” and future research avenues.

## Introduction

The notion of “personalized medicine” is often understood in relation to medical treatments tailored to individual needs and based on a pool of digitalized data pertaining to the biological, behavioral, social, and environmental determinants of health (Maughan, 2017; Prainsack, 2017). This notion mostly acts at and for an “individual” level (critically Juengst et al., 2012; Prainsack, 2018). However, there are signs for a *reconfiguration* of this notion of personalization. Certainly, the fact that professional, institutional, commercial and research practices have been opening participatory avenues to involve patients has long been part of the narrative of “personalized medicine” (Swan, 2009, 2012; Prainsack, 2017). However, new and more grassroots-driven approaches of health-related citizen science activities (Vayena et al., 2015) and health data governance (Blasimme et al., 2018) have emerged, reconfiguring boundaries between experts and ordinary people. We add that crises, such as the COVID-19 pandemic and environmental or climate disasters, may further accelerate this already ongoing blurring of divides between experts and ordinary people, changing the borders of institutional territories of knowledge. Thus, a new understanding of personalization might emerge, this time enacted “from below,” where *below* stands for deriving from/produced by ordinary people (i.e., the grassroots) but does not imply a hierarchy.

Given such a reconfiguration, the traditional understanding of personalization might need to embrace dimensions that go beyond an individual-centered view of a person's wellbeing, including more collective and *altruistic* understandings of services. Furthermore, traditional (health) services provision is generally still based on the expert/practitioner-layperson divide and on a rather “paternalistic” approach to the person that needs such services (Chiapperino and Tengland, 2015; Prainsack, 2017). We argue that a valorised inclusion of contributions from below in health services provision would imply reforming hierarchical structures dominating in “institutionalized” health-related practices. In addition, we posit that this reconfiguration demands a consistent commitment from institutional actors to embrace the contribution that ordinary people, well-organized and aware of their health needs, could bring.

In this perspective article, we explore practices that signal forms of “data altruism” aimed at personalizing health services to the needs of *unrepresented* and *neglected* groups. We regard these groups as those that do not feel sufficiently or at all included in the design and implementation of health services because their particular health conditions have been under-researched and/or neglected by policy-makers due to entrenched bias and structural forms of discrimination.

*Data altruism* is understood as the situation in which some people decide to voluntarily donate their personal data for a common goal such as scientific research. Health *services* include both those organized by the government or any other institutional body to the benefit of a particular society or community, and those that are “auto-organized” from below, by grassroots actors that complement or substitute “official” service provisions. We explore whether grassroots-driven data altruism strategies could make health services provision more inclusive of people's values and experiences, bringing in a form of “personalization from below.”^1^

As a start, we contextualize these practices as a specific form of science-based knowledge production which has been called personal health science elsewhere (Heyen et al., 2019). We then elaborate on some real cases and their data governance models, before we reflect on the implications of “personalization from below” for alternative public health interventions. We conclude by outlining future research avenues.

## “Personalization from below” as a form of personal health science

Whereas the vision of “personalized medicine” focuses on the core of the healthcare system, thus on medical treatments, there have been other developments in the context of personalized health that take place largely *outside* established medical or scientific institutions. Two very prominent examples are Direct-to-Consumer (DTC) genetic tests provided by private companies such as *23andMe*,^2^ and digital self-tracking *via* wearables and digital health apps. The promise of the commercially operated DTC services is to enable everyone (with a sufficient level of technology access/ability/understanding) to produce personalized knowledge about one's own body (e.g., *via* genetic tests). The promise of digital self-tracking tools is to enable individuals to obtain greater knowledge about their health status than they used to have in the past. Both cases have been the subject of numerous, also critical, analyses (e.g., Van Dijck and Poell, 2016; Sharon, 2017). As Juengst et al. (2012) argue, for instance, such practices often depict patient empowerment as “the solution” to an ever-present healthcare crisis, but at the same time risk to center responsibility for healthcare excessively on the patients. In any case, both examples represent activities and practices of science-based knowledge production which are initiated (in the case of DTC tests) or operated (in the case of self-tracking) by ordinary people and relate to their own personal health.

Heyen and Dickel (2019) have summarized these activities and practices under the term *personal health science*. Linguistically, Personal Health Science (PHS) is a term built by the authors through the coupling of three sub-terms: personal health, health sciences, and personal science. First, personal health refers to the individual health of one single person. The term suggests a personal view of one's own health and thus a (lay) perspective commonly distinguished from professionals and experts. Second, health sciences refer to the interdisciplinary field of professionally conducted research on human health. Finally, personal science means both research into one's own person and *a specific form of citizen science*, since it is usually laypersons and not professional scientists who become scientifically active and research themselves (Heyen, 2016, 2020; Senabre Hidalgo et al., 2022). Already on the basis of this simple conceptual chain, Heyen and Dickel (2019) argue, PHS can be located at the interface of health (or the healthcare system), science (or the science system), and society (or the public): laypersons research and care for their personal health. Thus, PHS practices always have both a scientific reference, since the knowledge production has at least a scientific or scientific-technical basis, and a self-reference, since the knowledge concerns the health of a concrete person engaged in these practices.

The phenomenon of “personalization from below” seems to represent a third type of PHS practices. It is neither about the commercially-driven and professional-based scientific analysis of personal health data for the benefit of science and the individual user or data donor (such as the DTC genetic tests and also platforms like *PatientsLikeMe*,^3^ representing a first type), nor is it about the pure individualistic practice of ordinary people doing research on one's own body and health without striving for any additional common good-oriented purpose (such as personal science, representing a second type). Instead, “personalization from below” in our framing:

- Is the scientific research on one's own health organized and led by civic organizations, patient groups, local communities, or even individual patients or laypersons;
- Aims at the production of knowledge that is both (potentially) generalizable for science and applicable for personal health purposes of the participants (or even for wider public health services); *and*
- Creates an added value for more collective or even altruistic purposes beyond commercial profit and one's personal health.

## Health data production and governance in “personalization from below”

Instances of “personalization from below” in the health sector can be found in data altruism initiatives and emerging data governance models. With data altruism we indicate the donation of (personal health) data for public interest purposes by single individuals, such as in citizen-science studies, art projects or civic-led initiatives. Data altruism differs from other forms of data generation, in which laypersons produce data about their health status (i.e., with self-tracking apps) and third parties get access to it according to the terms of service of the platform. It refers to data exchanges established explicitly for public interest purposes. Data subjects collect new data or share information with a third party for a public interest purpose, like for research and advocacy. For instance, data altruism initiatives have been launched to address structural gender-based discriminations in health research and to reconfigure healthcare *via* new forms of data collection, sharing and use. These are based on bottom-up participation, *via* donation of information about various aspects of personal health, from weight to menstrual cycle, in certain cases obtained through self-sampling kits. Initiated by different social actors, ranging from research institutions, artists, grassroots movements and civic organizations, these initiatives advocate for better and fairer healthcare for all (Salas Seoane et al., 2022).

An example is Isala,^4^ a citizen science project at the University of Antwerp, developed within the framework of the larger Lacto-Be project,^5^ which involved over 4,500 participants who provided detailed information about their health status and sent samples collected with vaginal swabs. The study allowed to increase understanding of the female microbiome, which is crucial for women's health and reproduction but whose ecology and determinants in the general population are still unclear with severe consequences on women wellbeing (Lebeer et al., 2022). A project with a similar goal is Transbiome,^6^ developed to provide a basic understanding of the vaginal microbiome for transwomen who undergo gender-affirming to surgeries, with the aim to fill the knowledge gap about transwomen's microbiome. A related topic has been addressed by Alma,^7^ an art project based on a participatory methodology. Women have been involved through the use of special sensors, for monitoring information in vaginal fluids, with the goal of creating an atlas of female intimate health and of helping those suffering from recurring gynecological conditions. Another relevant case is that of a participatory research conducted within the framework of the EU-funded project “TRANSFORM,”^8^ where women acted as co-researchers talking in first person about endometriosis in Catalonia, a matter on which they felt that their voice was under-represented. The initiative embraced citizen science methods to raise awareness on how endometriosis is experienced by the affected women, and produced first-person recommendations to improve diagnostic and care services (Salas Seoane et al., 2022). Overall, these initiatives are “altruistic” insofar as data collected/shared not only lead to better knowledge about the self for each participant, but also produces collective benefits, increasing knowledge on under-researched topics, and advocating for more research and better healthcare.

Instances of “personalization from below” in the health sector can also be found in an emerging model of data governance that enables collective control over data and its use: data cooperatives. Data cooperatives have been flourishing especially in the health sector to enable citizens to control their personal health information and donate for research, see for instance initiatives such as Salus.Coop,^9^ MiData^10^ and OpenHumans^11^ (Greshake Tzovaras et al., 2019). They allow individuals to exert direct control over their personal data, by aggregating information collected from multiple sources and integrating it with that of all members, to increase knowledge and purse collective goals that benefit members of the community and the wider society (Blasimme et al., 2018). Data cooperatives are part of a wider constellation of “alternative” data governance models, which contest the dominant logic of accumulation and extraction in the current data economy according to which data is merely a driver for economic growth (Mulgan and Straub, 2019; Micheli et al., 2020; Korjan and Narayan, 2021; Sadowski et al., 2021; UK AI Council, 2021). Mainly adopted by Big Tech and large companies, who collect data on their customers, the extractive logic is starting to permeate also States and public health authorities, that “no longer maintain a monopoly on large-scale data collection, but find themselves competing with businesses for a share of revenues to be extracted from data from the population” (Tupasela et al., 2020, p. 5).

Data cooperatives, instead, are a response from below to those trends, as they are led by civic actors (civic society organizations, citizens, informal groups), are based on different values (inclusion, equity, redistribution of value and public interest) and aim to reshape power relations around data control and value. At the moment, these are small scale and niche initiatives, yet, they are shaping the debate on how alternative governance approaches to (health) data might occur (Sandoval, 2019). In fact, not only data cooperatives are mentioned by a growing body of literature, but they are also supported by the EU Data Governance Act, a regulation included in the European Strategy for Data, which is meant to increase trust in data sharing fostering the establishment of neutral data intermediaries and data altruism.

Health data cooperatives are instantiations of what “personalization from below” could look like, as they stand in stark opposition, both in terms of scale, governance and values, to top-down initiatives by governments or big tech companies aimed at building large personal data repositories for research on personalized medicine (Blasimme et al., 2018). Not only they allow individuals to have control of their own health data, steering its use according to their motivations and concerns, but they also produce collective benefits through a more democratic governance approach: they are inspired by a political drive to increase the possibility to govern data from below. Members of data cooperatives are not just seeking individual benefits, they act as a community with shared interests and use data to satisfy collective interests (e.g., increased knowledge on a rare disease), which cannot be pursued individually (UK AI Council, 2021). Marginalized social groups and underserved communities can organize data cooperatives to make their voice heard, taking control of their data and influencing the direction of scientific activities, for instance redressing the under-representation of neglected communities in health research databases (Blasimme et al., 2018). Data cooperatives can offer access to aggregated data that did not existed before, for under-researched themes or on under-represented populations, which can have a transformative power for health public service delivery.

## Alternative public health approaches triggered by “personalization from below”

The illustrated experiences suggest three main arguments for how “personalization from below” can support alternative public health approaches. First, an epistemic argument, which is that knowledge of health issues can be overall improved by “personalization from below.” The data that people share in these initiatives are often shedding light on under-researched matters and come from the knowledge of under-represented groups. Furthermore, the data that people share are frequently enriched with people's values and their demands for a different way to imagine health services provision. By relying on such data, personalization approaches can represent more members of the population and the knowledge stemming from said practices becomes more generalizable.

The second is a democratic argument to favor “personalization from below,” as the political legitimacy of any public health intervention in any group can benefit from considering the initiatives that are manifestation of this trend and the related data produced. Indeed, when institutions manage to “embrace” the good of these practices, methodological and socio-political innovation can occur. Launching, joining or embracing an initiative can be regarded as a *political act* that inform policy discourses on public health promotion. The said practices may embody expressions of rights (for example, right to participation, to healthcare, to dignity, to representation) and of values (for example, respect for and inclusion of unrepresented and neglected communities and their understanding of services). Data stemming from such initiatives could help institutions in making services provision more attentive of different worldviews, re-shaping them in a way that is more centered on actual needs of specific communities. This could make services arguably more democratic.

A third aspect, connected to the first two, it is an equality argument in praise of “personalization from below.” Promoting the said practices can help making visible the issues, concerns, needs and health priorities of neglected groups. At present, this is found first and foremost in scientific research arenas. Synergies are indeed multiplying between researchers and social groups that feel “neglected” which demonstrates that researchers recognize the value of “personalization from below.” Just to highlight some European cases, in the previous section we mentioned the Isala project, an initiative deployed in the framework of a research project funded by the European Research Council, which engaged women as citizen scientists to advance the understanding of lactobacillus' beneficial potential for vaginal health. In other instances, neglected groups stood up on social media without the “mediation” of researchers. An example is offered by the movement for the recognition of Vulvodynia^12^ in Italy.^13^ The initiative adopted a bottom-up approach based on sharing of information and community-building on social media to increase public awareness of an illness perceived as largely ignored and misunderstood. By posting personal stories on social media, participants (both patients and doctors) shared health data and created a (digital) space for discussion (Pieri, 2022). Recently, the mobilization led to the first proposal for a law for the recognition of Vulvodynia as a medical condition.^14^

## Conclusion and future research

Our reflection, situated at the intersection of personalized health and knowledge co-production based on altruistic health data sharing, builds an alternative understanding of “personalization” that differentiates itself from a more individual-centered notion of what “personalized” means. We illustrated examples of grassroots-driven triggers to innovate health services provision. By contributing their data and time, people demonstrate that a certain matter is important to them because it is affecting them directly (e.g., a personal illness) or it is putting at risk values in which they believe (e.g., lack of recognition for the needs of an underserved group). Such flourishing small-scale initiatives shape the debate on health data governance and they shed light on under-represented health concerns or disparities that are not prioritized by policy or market agendas. They also push for regarding data as a common resource for the benefits of a (more or less extended) group of people, defining themselves how these benefits are understood and should be pursued. We speculate that in a near future people might increasingly shift from demanding data and services from “official” channels to openly providing data and even services that can be of value for institutions and other citizens. Institutions in charge of services provision should look at these practices as possible models of alternative public health interventions and design appropriate “policy uptake” strategies (Berti Suman, 2021).

Institutional support to these initiatives would entail a twin transition, pairing socially just interventions with data-driven innovation, and shaping both according to civic values. In addition, these practices will need regulation, validation and standardization to avoid abuses and misinformation masked under the vests of “good data.” Such task could be performed by gate-keeping actors and stewards, which could be practitioners, researchers and research institutions, and civil society's associations. The role of institutions in the field could be to oversee the quality of data collected by the grassroots groups, and to promote digital literacy and equal access for disadvantaged communities. The question on how can these communities' values, demands, and imaginaries be embedded into data and how can governance models accommodate this in a way that they translate into services is still open. However, we believe that institutional support to scale up successful, but still niche, civic experiences could enable or at least facilitate this outcome. This implies challenging hierarchical structures often dominating healthcare, and adopting concrete interventions to embrace the contribution from small scale grassroots initiatives and help them scaling up.

In this brief Perspective, we could not fully grasp the epistemic, organizational, legal, regulatory, and political heterogeneity of the discussed developments. We also could not make justice to the diversity of social actors (e.g., civic associations, activists, patients, healthcare professionals, and policy-makers) that play a role in the field. In this fascinating, yet still largely unexplored field we deem that further research is needed along the following lines, among others. Empirical research should review scenarios—such as crises and disasters—that can act as enabling factors spurring “personalization from below.” Furthermore, inquiry is needed to explore which values, demands and rights' claims people embed in the data they produce. Investigation on how public actors can make wise use of them through a benefit sharing approach could be useful. Research should also assess more in-depth the ruptures and continuities with traditional personalized health approaches. Comparative case study analyses could help refine and describe the said model(s) and approach(es), and assess their impacts on service provision in specific domains and contexts. A comparative investigation can also shed light on the values traditionally present in initiatives that are manifestation of “personalization from below,” compared to those typical of personalized medicine initiatives. Legal implications should be explored, for example, regarding the potential risks for privacy and data protection of the participants and the likelihood of market capture, especially when there are hidden interests to profit (Berti Suman and Pierce, 2018). Answering these and further questions will be pivotal to shape agile and just health services provision in the near future. We hope our Perspective added a viewpoint and a step ahead in this direction.

## Data availability statement

The original contributions presented in the study are included in the article, further inquiries can be directed to the corresponding author.

## Author contributions

All authors listed have made a substantial, direct, and intellectual contribution to the work and approved it for publication.

## Footnotes

^1^Prainsack (2017, p. 11) in her work used the term “personalization from below,” yet with a different understanding than what embraced in our contribution. In our opinion, she refers to the general data contribution and other efforts of patients within the paradigm of personalized medicine. In contrast, our proposition orients this concept towards “neglected” groups operating from below that—through contributing health data—demand to make (health) services provision more inclusive of values that matter to people.

^2^See https://www.23andme.com/.

^3^See https://www.patientslikeme.com/.

^4^See https://isala.be/.

^5^See https://cordis.europa.eu/project/id/852600.

^6^See https://www.transbiome.org/.

^7^See https://al-ma.org/Smart-Underwear.

^8^See https://www.transform-project.eu/transforming-the-patients-experience-through-citizen-science/.

^9^See https://www.saluscoop.org/.

^10^See https://www.midata.coop/en/home/.

^11^See https://www.openhumans.org/.

^12^Vulvodynia is a persistent, unexplained pain in the female genital area which can become a long-term and very distressing ailment.

^13^See https://www.vulvodinianeuropatiapudendo.it/.

^14^See https://www.quotidianosanita.it/governo-e-parlamento/articolo.php?articolo_id=104439.
